# Non-Tuberculous Mycobacteria in TB-Endemic Countries: Are We Neglecting the Danger?

**DOI:** 10.1371/journal.pntd.0000615

**Published:** 2010-04-27

**Authors:** Krishnamoorthy Gopinath, Sarman Singh

**Affiliations:** Division of Clinical Microbiology, Department of Laboratory Medicine, All India Institute of Medical Sciences, New Delhi, India; Kwame Nkrumah University of Science and Technology (KNUST) School of Medical Sciences, Ghana

## Introduction

There are more than 120 members of the genus Mycobacterium, which are diverse in pathogenicity, in vivo adaptation, virulence, response to drugs, and growth characteristics. Mycobacteria other than *M. tuberculosis* complex and *M. leprosy* are known as Non-Tuberculous Mycobacteria (NTM) and are known by various acronyms. They attracted abrupt attention only after the AIDS epidemic, but most of the reports were published from TB non-endemic countries [1] and only rarely from TB-endemic countries. This is probably because the chances of missing NTM species are higher in TB-endemic countries, which are poorly equipped and overburdened with other diseases (Box 1). The information regarding their true incidence and prevalence in these countries is scarce [2]. In the absence of such authentic information, the current dogma has been that the NTM are of the least consequence. However, we do not agree with this myth and wish to present our viewpoint on this important aspect and emphasize the need for a fresh look at this neglected aspect.

**Box 1. Possible Factors for Under-reporting of NTM from TB-Endemic Countries**

- NTM infections are not reportable in any country.
- Awareness is lacking among treating physicians and microbiologists.
- Laboratory infrastructure is lacking for culture and identification of non-tuberculous mycobacteria.
- High burden of TB and HIV attracts the bulk of the attention of the health care system; governmental fiscal inputs toward the costs of these neglected infections continue to be neglected.
- Standardized or accepted criteria to define NTM respiratory disease are lacking.

## Prevalence of NTM Infections before and after the AIDS Epidemic

We searched methodological search terms and phrases such as “non-tuberculous mycobacteria and AIDS” in Medline records and found that 3,020 articles were published between 1981 and 2009. Using the same phrase, only 59 articles were published between 1900 and 1981, indicating a clear upsurge of NTM disease in the post-AIDS era. However, most of these publications were from TB non-endemic countries [1]–[5], but not much significance could be adhered to these isolations [2]. The disseminated NTM infection is typically seen when the CD_4_^+^ T lymphocyte number falls below 50 µl. For this reason, it is argued that in TB-HIV co-endemic countries, AIDS patients usually die of tuberculosis or other infections before their CD_4_^+^ count falls low enough for NTM to cause a disease (Box 2). Nevertheless, we feel that besides this argument, in a majority of the patients, the diagnosis of NTM disease gets missed in these countries.

**Box 2. Facts about Non-tuberculous Mycobacterial Disease**

- AIDS patients are significantly more vulnerable to NTM infections due to severe T cell immunodeficiency.
- Solid organ transplant patients, even though immunocompromised, are not at as high a risk as their HIV-positive counterparts.
- Although some genetic and anatomical factors predispose to NTM, no proven associations have been proven among geographical, occupational, or ethnic factors and NTM infections.
- Anatomical abnormalities and other co-morbidities such as chronic obstructive pulmonary disease (COPD), bronchiectasis, cystic fibrosis (CF), pneumoconiosis, past history of TB, pulmonary alveolar proteinosis, and esophageal motility disorders are well-established predisposing conditions.
- Disseminated NTM infections have been associated with specific genetic syndromes such as mutations in interferon (IFN)-γ, interleukin (IL)-12 synthesis, and in response pathways and the nuclear factor-κB essential modulator (NEMO).
- Conventional methods are not sufficiently sensitive to estimate prevalence and incidence of NTM infections.
- Monoplex TB-specific PCR needs to be replaced by multiplex PCR systems on relevant clinical samples along with blood and urine samples in tertiary-care settings.
- Multiplex PCR primers have been designed to amplify genus-specific regions, *M. tuberculosis* complex specific, *M. avium* complex specific, *M fortuitum* complex specific, and species-specific gene targets, that can be performed in a single tube.

## Geographical Distribution of NTM

Some workers also consider that the low detection rate of NTM is due to diversity in the environmental and climatic conditions in the HIV-TB-endemic countries, but this argument is not supported by the literature [3]–[5]. In most of the surveys, the rate of human NTM infections is estimated by non-specific antibody assays or skin tests [6]. Hence, these findings may not be a reliable source of information. The International Union against Tuberculosis and Lung Diseases (IUATLD) reviewed data from 14 countries and found that the *M. avium* complex (MAC) was the most frequently isolated species in all these countries, which included China, India, and Korea. While *M. fortuitum* was the most frequently encountered species in Belgium (2.1%), the Czech Republic (17.5%), Denmark (5.3%), Finland (6.7%), France (6.5%), Germany (12.2%), Italy (2.5%), Portugal (16.5%), Spain (10.8%), Switzerland (17.5%), Turkey (33.9%), and the United Kingdom (6.0%), undoubtedly, environment is the main reservoir of NTM. There is no evidence of human-to-human or animal-to-human transmission [6]. Most infections are acquired either from the water (treated or untreated) or soil. MAC and *M. fortuitum* are frequently isolated from the drinking water distribution systems and swimming pools in both developing and developed countries.

## Overlapping Disease Manifestations: Reason for Underreporting

The overlapping clinical manifestations of the diseases caused by *M. tuberculosis* make the specific diagnosis of NTM difficult. Even though fever is less common in NTM pulmonary disease, most patients will present in a chest x-ray with sporadic infiltrations, nodular lesions, and cavities indistinguishable from pulmonary tuberculosis, and disease caused by *M. kansasii* in HIV-infected patients often mimics pulmonary tuberculosis [6], [7], [8], [9]. *Mycobacterium avium-intracellulare* commonly causes disseminated diseases in AIDS patients. Other NTM infections also produce highly non-specific manifestations including reactivity to a PPD skin test [6], [8]. Further, these manifestations are often overshadowed by co-morbidities such as chronic obstructive pulmonary disease (COPD), cystic fibrosis, defects in the chest wall, gastroesophageal reflux diseases, bronchiectasis, aspergilloma, etc. [6]. Albeit in pulmonary NTM disease chronic cough and fatigue are very common, fever and sweats may be less frequent, unlike tuberculosis. Malaise, hemoptysis, weight loss, and wasting are uncommon and usually indicate advanced disease. NTM co-infections with *M. tuberculosis* disease are not infrequent but are rarely diagnosed [2]. The NTM infections pose a challenge for a directly observed treatment–short course (DOTS) programme because under this programme the patient is treated only on the basis of smear findings.

## Ethnicity, Occupation, and Genetic Susceptibility?

In a recent study carried out in Thailand [7], it was found that nearly half (46%) of the disseminated NTM infections in HIV-negative cases were associated with farming. The commonest organ involved was lymph node (89%), followed by skin and soft tissue (26%). The agricultural injuries added with exposure to NTM contaminated soil and water lead to tissue invasion and disease causation. Some authors have reported the role of genetic susceptibility for NTM infections, such as the multiple mutations in the interferon-γ receptor 1 gene [10] and with parental consanguinity. However, consanguinity is not confined to any particular ethnic group or geographic region. In one study Caucasian ethnicity was correlated with NTM lymphadenitis and non-Caucasian ethnicity with tuberculosis [11]. Ethnicity is proposed as the reason for low prevalence of NTM in TB-endemic countries by some workers. We, however, think that it is instead due to the prior exposure of this population to *M. tuberculosis*, which mounts an adaptive protective immunity against NTM. Studies have also shown a vice versa effect, that adaptive immunity developed by exposure to NTM can provide cross-protection against TB and leprosy [12]. Therefore, we feel that ethnicity is least likely to be the reason for this underreporting. In our opinion, NTM are most likely being missed due to lack of awareness. In India, we were probably the first to highlight this issue, which is hesitantly but steadily being appreciated by others now.

## Association of NTM with Other Diseases

Normally most NTM infections are asymptomatic, but under certain circumstances these mycobacteria can cause a variety of symptoms, including serious morbidity and mortality. For instance, the strongest association of NTM pulmonary infections has been reported with structural lung disease, such as COPD, bronchiectasis, cystic fibrosis, pneumoconiosis, pulmonary alveolar proteinosis, and esophageal motility disorders [6]. Though disseminated NTM infections typically occur only during severe immune suppression, in HIV-negative patients disseminated NTM infections are rare and have been associated with specific genetic syndromes such as mutations in interferon (IFN)-γ and interleukin (IL)-12 synthesis and defects in response pathways including the signal transducer and activator of transcription 1 (STAT1) and in the nuclear factor-κβ essential modulator (NEMO]. Often these genetic defects are associated with an increased susceptibility to various opportunistic infections and hypersensitivity responses. All mycobacteria including the NTM are strong inducers of Th-1 immune responses, and this immunomodulatory effect may lead to hypersensitivity reactions, instead of beneficial effects. It is shown that a new subset of lymphocytes (Th17) is triggered by BCG, which induces the production of pro-inflammatory cytokine (IL-17), which is considered responsible for several autoimmune diseases including asthma, hypersensitivity pneumonitis, allergic airway inflammation, diabetes mellitus, etc. [13]. Another NTM, *M. avium* ss *paratuberculosis* (MAP), is a well-established cause of Johne's disease in animals, but its direct role in the etiology of Crohn's diseases has been opposed [14]. Most of these studies have shown only an association, and the disease is considered an immune-mediated condition where some genes increase susceptibility of the gut mucosa to bacterial flora including the MAP. One such gene is *CARD15/NOD2* situated within the IBD1 region of chromosome 16q12. Greater discussion on this issue is out of the scope of this article.

## Diagnosis

**Figure 1:**
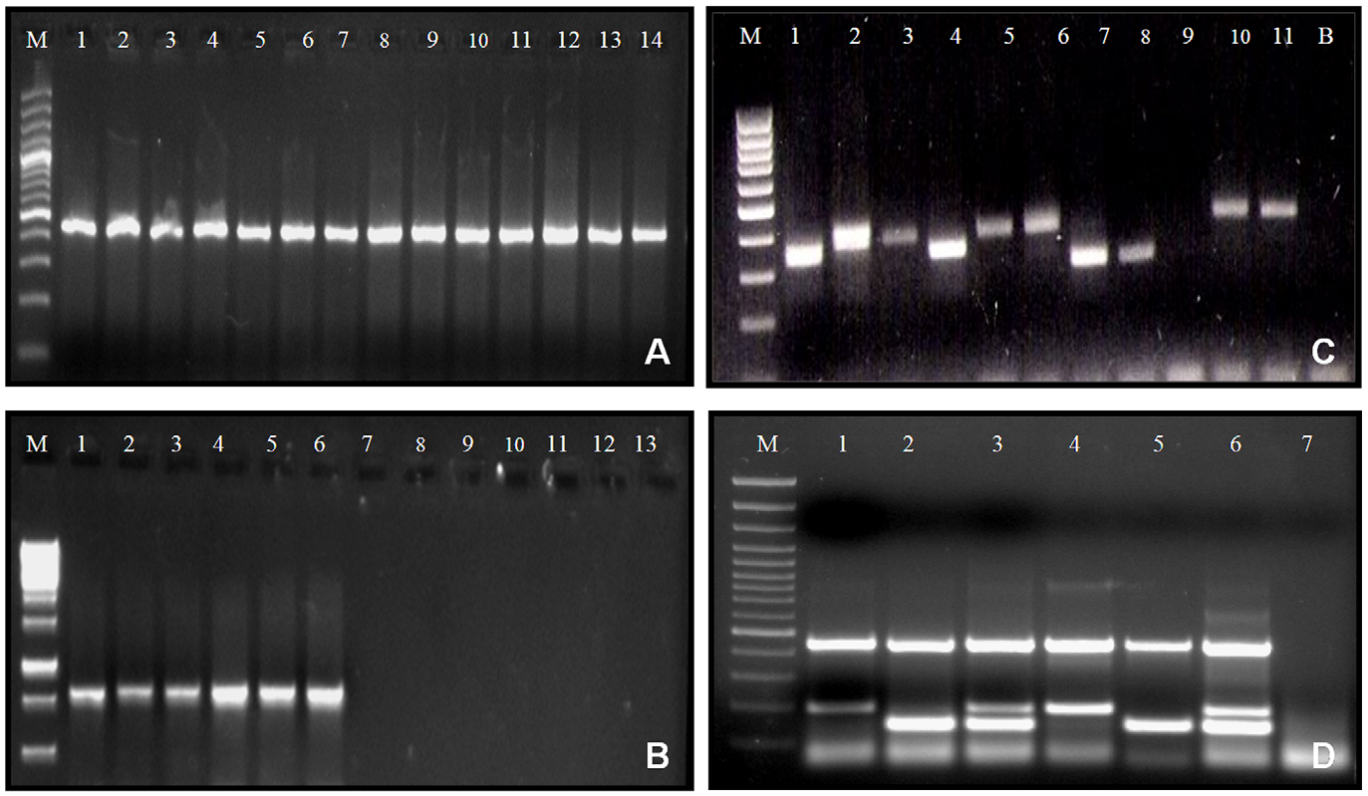
Genus- and species-specific diagnoses can be accomplished by using various PCR systems. (A) Genus-specific hsp65 PCR. From lane M–14 are 100 bp marker, *M tuberculosis*, *M. terrae*, *M. scrofulaceum*, *M. fortuitum*, *M. duvalii*, *M. smegmatis*, *M. chelonae*, *M. celatum*, *M. bovis* (BCG), *M. kansasii*, *M. bovis*, *M. flavescens*, *M. goodii*, and *M. avium*, respectively. Note a 441 bp amplicon in all species. (B) TB complex-specific cfp32 PCR. From lane M–14 are 1,000 bp marker, *M tuberculosis* H37 Rv, *M tuberculosis* clinical isolates (3), *M. bovis* (BCG), *M. bovis*, *M. scrofulaceum*, *M. fortuitum*, *M. duvalii*, *M. smegmatis*, *M. chelonae*, *M. celatum*, *M. kansasii*, and *M. flavescens*, respectively. Note a 786 bp amplicon in *M. tuberculosis* (3) and *M. bovis* (2) species. (C) PCR amplification of 16–23 S-rRNA using genus-specific primers ITS-F and Mycom-2; Lane M, 100 bp Marker; Lanes 1, 4, 7, and 8 are *M. tuberculosis* isolates (121 bp); Lanes 2, 3, 5, and 6 are MAC isolates (144 bp); Lanes 9 and 12 are negative controls; and Lanes 10 and 11 are *M. fortuitum* isolates (223 bp). Other species of mycobacteria also yield amplicons of variable size specific for each species (unpublished data). (D) A multiplex PCR comprised of primers for Mycobacterium genus (targeting hsp65), *M. tuberculosis* (targeting cfp10), and *M. avium* complex (targeting MAC) specific genes. Lane M is 100 bp marker, Lanes 1 and 4 show *M. tuberculosis* single species infection (441 bp and 191 bp bands), Lanes 2 and 5 show *M. avium* complex single infection (441 bp and 144 bp bands), while Lanes 3 and 6 show mixed infection of *M. tuberculosis + M. avium* complex (3 bands of 441 bp, 191 bp, and 144 bp size). The last lane is negative control.

The major reasons for under-diagnosis of NTM in TB-endemic regions are lack of awareness, limited laboratory facilities, and overburden of other diseases. Under the DOTS programme, treatment of pulmonary tuberculosis is started only on the basis of sputum microscopy results, which has an inherent possibility of missing the NTM disease [15]. While most of the laboratories in TB-endemic countries are dependent only on smear examination to make the diagnosis of tuberculosis, experience shows that application of newer diagnostic methods such as rapid culture methods, multiplex PCRs, DNA probes, and/or 16S rDNA sequencing methods is more rewarding [2]. In our settings, we found NTM in 17.6% of the suspected MDR-PTB cases and in 12.4% of the suspected extra pulmonary tuberculosis cases when we applied molecular methods [2], [9]. Therefore, to obtain the best results, it is recommended that in addition to high-quality sample collection, the cultures should be inoculated in both liquid and solid egg-based media and preferably incubated at different temperatures (at 28°C, 37°C, and 45°C). The Multiplex PCR developed in our setting directly on clinical samples was highly useful in detecting the single and/or co-infections of *M. avium* and *M. tuberculosis* [2]. Liquid cultures are also useful in the isolation of NTM from blood samples of AIDS patients. The conventional biochemical and phenotypic methods are tedious, less rewarding, and take a long time to speciate the mycobacterial isolates. The application of PCRs, DNA probes, and/or DNA sequencing methods makes the characterization of NTM species the least ambiguous. Recently, excretory proteins such as MPB64 and MPT63 have shown potential for differentiating MTB and NTM with high accuracy [16]. Therefore, it may be concluded that multiplex PCR systems targeting multiple genes such as 16S-rRNA, hsp-65, ESAT-6, *MAC*, cfp-10, or the internal transcribed spacer (ITS) region of 16–23 S rRNA primers are highly precise, rapid, cost-effective, and can be used directly on clinical samples (Figure 1) [2].

## Treatment

Most of the NTM except *M. kansasii* are inherently resistant or partially susceptible to the standard anti-tubercular drugs. Nonetheless, the availability of newer macrolides/azilides has drastically changed this situation. The macrolides clarithromycin and azithromycin have become the cornerstones of therapy for MAC [6]. Hence the utility of in vitro drug susceptibility testing for NTM is an important laboratory support. We have recently standardized the in vitro drug susceptibility testing for most of the NTM isolates. Most of the NTM we studied were susceptible to Ofloxacin (98%) and ciprofloxacin (90%), but *M. mucogenicum* was susceptible only to clarithromycin [9]. For rapid growers, testing should be done against amikacin, clarithromycin, quinolones, sulfamethoxazole, doxycycline, and imipenem. Linezolid and tigecycline may also be active. Localized disease, especially with difficult-to-treat NTM, may benefit from surgical resection usually with adjunctive antibiotic coverage of the NTM. Other complications of NTM infection need to be treated symptomatically, including the use of corticosteroids, if warranted.

## Conclusion and the Way Forward

Even though there is no significant difference in the incidence, prevalence, and distribution of clinically relevant NTM among various geographical or ethnic subsets of countries, lack of awareness and limited laboratory facilities are the underlying reasons for their underreporting in TB-endemic countries. Several risk factors have now been identified, and it is possible to minimize the NTM disease in these vulnerable patients. For species-specific identification, it is recommended that networks of national and regional laboratories be developed. The conventional methods of speciation must be discouraged due to their non-reproducibility and tediousness. Molecular methods such as multiplex PCR protocols need to be put in place. Recognition of NTM as an “emerging pathogen” would perhaps elevate the status of NTM for better research funding. Also, development of an animal model would accelerate our understanding of NTM disease and their pathogenesis (Box 3).

**Box 3. Summary and Conclusions**

The infections due to non-tuberculous mycobacteria (NTM) are increasing worldwide, detrimentally affecting both HIV seronegative and immunocompromised individuals. Cases of non-tuberculous mycobacteria are reported mainly from European countries and America, where tuberculosis is not endemic. In TB-endemic regions such as Southeast Asia and sub-Saharan Africa, the occurrence of NTM is under-reported. This article reviews the various possible hypotheses of reportedly low incidence and prevalence of NTM in TB-endemic countries, and the authors provide their own view point on this issue. The authors think that:

1. There is a lack of systemic reporting of non-tuberculous mycobacterial diseases due to overlapping clinical manifestations of tuberculosis and NTM diseases.
2. There is a lack of proper infrastructure for the identification of non-tuberculous mycobacteria in TB-endemic regions.
3. Because of the high burden of tuberculosis in these regions, the whole attention of health care workers and government is directed toward TB.
4. There is no geographical area or country unfit for the survival and spread of NTM.
5. The notion that some ethnic groups or races are inherently resistant to NTM infection has not been scientifically proven.
6. There is sufficient evidence that non-specific cross-immunity is developed due to latent tuberculosis against these less-virulent NTM.
7. There are no systemic regional surveys for evaluating the true prevalence of NTM.
8. Empirical use of fluoroquinolones and anti-tubercular drugs in relatively dysfunctional health care settings gives a false impression of a low incidence of non-tubercular cases.
